# 

**Published:** 1930

**Authors:** 


					Vol. XLVII. No. 175. SPRING, 1930.
Hnt i 13P5 t* - U "* ' *; :v> ' ??
- -
I.. "7'
.
I?. ?JI
dico-Chirurgical Journal
-sMrl
PUBLISHED UNDER THE AUSPICES OF
THE BRISTOL MEDICO-CHIRXJRGICAL SOCIETY.
?? *, ?"? - - ; . ' ' ' .
Editor: J. A. NIXON, C.M.G., M.D., F.R.C.E, ^
?;.... ^vVorary of /^s
WITH WHOM ABE ASSOCIATED JSZ)*
' O %
E. W. HEY GROVES, M.S., F.R.O.S., B.Sf,
E. WATSON-WILLIAMS, M.O., Oh.M., F.R.G.sfcE.,
A. E. ILES, O.B.E., M.B., B.S.Load., Mudfe*
' -JL-? r *. *? ^ ' " "? ??+ rv< ?Tt
OAREY F. COOMBS, M.D., F.R.O.P., Assistant Editof
; / ... ?-grggg ?;?? * '? W -;V ?? j? . B
EditorM Secretary: A. L. FLEMMING, M3., Oh.B.
' ?
... ' ? '
-? ? - ' - ? , ?? ??? ; - - -
BRISTOL: J. W. ARROWSMITH LTD.. 11 QUAY STREET.
London : J. W. Akrowsmith (London) Ltd., 57 Gowsr Street, W.O.
* " :
I BP#^^KK8iS
saswar-i
***"'' ~ *J?
Price Three Shillings net.
^ " ** - ,
"

				

